# Are the Forces and Lower Limb Kinematics Displayed During Running Associated with Medial Tibial Stress Syndrome? A Case-Control and Case Study

**DOI:** 10.3390/jfmk11020214

**Published:** 2026-05-28

**Authors:** Joshua P. M. Mattock, Julie R. Steele, Karen J. Mickle

**Affiliations:** 1 Biomechanics Research Laboratory, School of Medical, Indigenous & Health Sciences, Faculty of Science, Medicine & Health, University of Wollongong, Wollongong 2522, Australia; 2 Global Sport and Movement Collaborative, School of Biomedical Science & Pharmacy, College of Health, Medicine and Wellbeing, University of Newcastle, Newcastle 2308, Australia

**Keywords:** shin splints, leg injuries, tibia, injury prevention, running biomechanics

## Abstract

**Objectives**: The aim of this paper is to determine whether leg kinematics and the normal force generated during the stance phase of running differed between (i) long-distance runners with medial tibial stress syndrome (MTSS) or (ii) long-distance runners who were asymptomatic at baseline testing but developed MTSS compared to asymptomatic control participants. **Methods**: Lower-limb kinematics, normalised stance-phase forces and spatiotemporal outcome variables were compared between the limbs of MTSS symptomatic long-distance runners (*n* = 11) and matched asymptomatic controls (*n* = 11). Outcome variables were also compared between the limbs of long-distance runners who were asymptomatic at baseline but developed MTSS (*n* = 4) compared to asymptomatic control limbs. **Results**: In the case-control comparison, MTSS symptomatic participants demonstrated slower running speeds but no differences in stance-phase normal forces or kinematics compared to asymptomatic controls. In the case study, participants who developed MTSS during the study displayed substantially lower normal forces, less plantar flexion and a more vertical tibia than the asymptomatic controls. **Conclusions**: The slower running speeds observed among the MTSS symptomatic participants may be pain-related or reflect reduced plantar flexor propulsive capacity. The development of MTSS by Participants 1 and 2, despite lower normal forces and plantar flexion compared with asymptomatic controls, suggests that the tibial load tolerance may vary among individuals. Furthermore, the peak stance-phase force appears to have limited utility as a standalone screening tool for MTSS injury risk. Finally, further prospective research is required to investigate plantar flexor function, propulsive force capacity and the risk of MTSS development to substantiate these findings.

## 1. Introduction

Medial tibial stress syndrome (MTSS) is an exercise-induced leg injury that frequently affects military personnel and athletes who undertake high training volumes that involve repetitive tibial loading, such as running [1]. Although the pathogenesis of MTSS remains uncertain, based on current evidence, it is hypothesised that tibial bone bending and a subsequent bone stress reaction of the tibial cortex beyond its remodelling capacity are the most likely causes [1].

During locomotion, peak tibial bone load coincides with the active peak force and results from internal muscle forces, external ground reaction forces and the global orientation of the leg [2,3,4]. Current evidence from in vivo and inverse dynamics studies indicates that in healthy individuals, posterior compressive stress and tibial torsion are the predominant loading patterns during stance [5,6]. Despite their value, both in vivo and inverse dynamic methods remain limited in clinical use due to invasiveness and computational complexity.

During the stance phase of gait, an individual’s leg alignment relative to the resultant ground reaction force vector can influence the sagittal plane bending moment of the tibia and the magnitude of tibial loading experienced [3,7]. Sasimontonkul et al. [3] hypothesised that running technique modifications aimed at achieving a more vertical tibial position during midstance and reducing the vertical ground reaction force active peak could reduce tibial loading and tibial stress fracture development. Although MTSS and tibial stress fracture are different pathologies, both injuries are thought to be associated with tibial loading above its remodelling capacity [1,8].

Although the external ground reaction force is not equivalent to the magnitude of tibial bone load [2,5], understanding the kinematics and kinetics during the stance phase of gait has the potential to provide insight into tibial bone loading when direct measurements or the modelling of tibial bone load are not feasible. During level running at progressively faster speeds, Matijevich et al. [2] reported a strong positive inter-subject correlation between the vertical ground reaction force active peak and peak tibial bone load (r = 0.97). Furthermore, Yang et al. [5] reported that an increased vertical ground reaction force and vertical free moment (torque about the vertical axis through the centre of pressure of the ground reaction force) were correlated with increases in anterior–posterior bending and tibial torsion, respectively. Lower limb kinematics have been implicated in the development of MTSS in long-distance runners [9,10,11,12]. For example, in a cross-sectional study, Becker et al. [12] reported that compared with matched control distance runners, individuals with MTSS displayed significantly greater rearfoot eversion at heel-off (*p* < 0.001) but no significant differences in the peak anterior–posterior propulsive force or peak vertical force during running. Furthermore, in the only prospective study to investigate the association between lower-limb kinematics in long-distance runners and MTSS development, Becker et al. [9] assessed pelvic, hip, knee and foot motion during running and plantar pressures while walking. They concluded that during stance, runners who developed MTSS demonstrated significantly greater contralateral pelvic drop (*p* = 0.021), rearfoot eversion (*p* = 0.017) and higher plantar pressures on the medial side of their feet at first foot contact and forefoot flat (*p* < 0.001–0.001). More recently, the authors of a case-control study of five long-distance runners with a history of MTSS and eight matched controls found that a greater rearfoot eversion moment persisted in individuals with a history of MTSS once symptoms resolved [13]. Previous kinematic and kinetic assessments of long-distance runners have predominantly investigated maximum joint angles, range of motion and peak forces, which may or may not coincide with peak tibial bone load. Furthermore, current MTSS research in long-distance runners is limited to assessing peak stance-phase ground contact forces during running through case–control studies or prospective evaluations of mediolateral plantar pressure ratios obtained during walking.

Peak joint angles and forces provide a snapshot of the kinematics and kinetics of individuals with and without MTSS. However, there is a dearth of research that has prospectively assessed running kinematics and ground contact forces using continuous comparison methods, such as statistical parametric mapping (SPM), to understand how these factors may contribute to MTSS development in long-distance runners. In one of the few studies to utilise this approach in an MTSS-symptomatic population, Langley et al. [14] compared tibia and rearfoot coordination variability in the frontal plane between recreational runners with MTSS and asymptomatic controls. The authors concluded that MTSS symptomatic runners exhibited greater antiphase movement of the tibia and rearfoot, which could increase torsional stress on the tibia. Although the authors assessed kinematics during the stance phase, the study is retrospective and limited to a single plane of motion. Therefore, further multiplanar prospective research is required to determine whether long-distance runners who develop MTSS display differences in lower-limb kinematics and the forces generated during the stance phase of running compared with their counterparts who do not develop MTSS.

The primary aim of this paper was to determine whether lower-limb kinematics and the normal force generated during the stance phase of running differed between long-distance runners with MTSS who abstained from rest and asymptomatic matched controls. The secondary aim of this paper was to determine whether long-distance runners who were asymptomatic at baseline testing and developed MTSS during the study displayed differences in lower-limb kinematics and the normal force generated during the stance phase of running compared with asymptomatic control participants. By combining cross-sectional, case–control comparisons with a prospective case-study approach, this paper aimed to characterise the biomechanical differences associated with both the presence of MTSS and its subsequent development, thereby providing complementary insight into potential injury-related loading patterns. We hypothesised that runners with MTSS would display less vertical leg alignment and greater peak force during the stance phase of running than matched controls. Furthermore, we hypothesised that runners who developed MTSS during the study would display less vertical leg alignment and greater peak force during the stance phase of running at baseline than asymptomatic control participants.

## 2. Materials and Methods

### 2.1. Participants

The participants in this paper were part of a larger prospective study conducted by Mattock et al. [15], which enrolled asymptomatic long-distance runners and long-distance runners with MTSS but who were actively training (i.e., abstaining from rest due to pain associated with their MTSS) (see Appendix A). Participants were included in the study if they were aged over 18 years, ran at least an average of 30 km per week for no less than 6 months, or were training for a long-distance event of at least a half-marathon distance. Asymptomatic long-distance runners were assessed at baseline and then followed to monitor their training load and for MTSS development. Participants suspected of having MTSS either at baseline or during the follow-up period were assessed by an experienced podiatrist [J.P.M.M] using the diagnostic criteria of Yates and White [16] to confirm the diagnosis. From the cohort of 63 long-distance runners (MTSS symptomatic = 11, asymptomatic = 52), 11 MTSS symptomatic individuals were matched on sex, age, height, body mass, weekly running training distance and limb dominance with 11 asymptomatic controls from the group of 43 runners who completed 12 months of running free from MTSS symptoms (see Table 1). Limb dominance was determined by observing which foot participants led with when stepping off a box [15]. Nine of the 11 MTSS symptomatic individuals experienced bilateral symptoms (average duration 14.8 ± 9.8 months). In the case of bilateral symptoms, a participant’s most symptomatic leg was determined using a numeric rating scale, and the corresponding matched control limb was compared to meet the assumption of data independence [17]. This provided a total of 11 symptomatic and 11 matched control limbs. Of the 52 asymptomatic participants at baseline, 2 developed bilateral MTSS (Participants 1 and 2; providing four limbs for analysis), and 7 participants were lost to follow-up (see Appendix A). Table 1 lists the characteristics of the MTSS symptomatic, asymptomatic matched controls and two participants who developed MTSS (Participants 1 and 2).

### 2.2. Treadmill Running Protocol

Participants ran on a SportsArt Fitness treadmill (Tainan, Taiwan) for 5 min at a self-selected running speed, which is equivalent to the speed estimated to attain their most recent 10 km race time. All participants were habitual overground runners; therefore, the first four minutes of the trial were used to familiarise participants with the treadmill, allowing them to achieve their natural running style. Data were collected during the final one minute of the running trial while participants wore the shoes in which they completed most of their weekly training (based on distance). The self-selected treadmill running speed was recorded for later analysis.

### 2.3. Forces Generated During Running and Cadence

The normal forces generated by each participant during the running trial were recorded (100 Hz) using Pedar-X insoles (Novel_gmbh_, Munich, Germany). Initial contact and toe-off (0–100% stance phase) were then determined when the normal force exceeded the baseline plus five times the standard deviation of the baseline noise for 10 steps [18]. Force data were normalised by the respective participant’s body weight (BW). Cadence was calculated as the number of steps per minute, and the foot strike pattern was determined using the strike index [19].

### 2.4. Lower-Limb Kinematics

Each participant’s lower-limb kinematics were tracked during the running trial using three OptoTRAK Certus^®^ Position Sensors (Northern Digital Inc., Waterloo, ON, Canada), sampling at 100 Hz. Thirty-eight smart markers were attached directly to each participant’s skin (double-sided toupee tape, Creative Hair Products, Melbourne, Vic, Australia) at specific bony landmarks on the pelvis, thigh and leg segments [15]. Foot markers were placed on each participant’s shoes directly over the bony landmarks identified by palpation. The three-dimensional coordinates of each marker during the last minute of each running trial were time-synchronised with the force data using a Pedar-X sync box (Novel_gmbh_, Munich, Germany).

The positional marker data were interpolated using a third-order polynomial least-squares fit algorithm, allowing a maximum of 10 frames for gap filling [20], and subsequently low-pass filtered using a fourth-order Butterworth digital filter (f_c_ = 15 Hz) [21]. Hip, knee and ankle joint angles were defined as the orientation of the distal segment relative to the proximal segment at each respective joint. Tibial alignment was defined as the orientation of the tibia relative to the vertical laboratory coordinate system where a positive value indicated forward tibial alignment (i.e., knee in front of ankle) and a negative value indicated backward tibial alignment. The kinematic variables were averaged over 10 steps for each participant’s limbs.

### 2.5. Statistical Analysis

Lower-limb kinematics (ankle, tibia, knee and hip) and stance-phase normal forces during running were compared between the 11 MTSS-symptomatic and 11 matched control limbs. All kinematics and normal forces were linearly time-normalised to 101 points representing 0–100% of stance. Normalised data were analysed using custom-made MATLAB (version R2022b) scripts. One-dimensional statistical parametric mapping was used to determine differences between groups, which were implemented using the SPM1D toolbox. To account for multiple comparisons, a Bonferroni correction was applied by dividing α = 0.05 by the number of comparisons. This yielded a corrected significance threshold of *p* < 0.0385 [22,23]. Data normality was assessed using the toolbox’s Shapiro–Wilk test and residual plots. Case-control comparisons were evaluated using one-dimensional SPM tests (two-sample) across the entire stance trajectory. For the case-study comparisons, an exploratory descriptive analysis of mean kinematic trajectories and 95% confidence intervals was conducted on baseline data from the dominant and non-dominant limbs of Participants 1 and 2, compared with the asymptomatic control limbs, because SPM methods are inappropriate for individual participant comparisons.

To explore whether observed group differences were influenced by running speed, post hoc SPM1D regression analyses were performed, including running speed, group and a speed × group interaction. This analysis was applied to the case-control comparison only when between-participant variability in running speed was present. For the comparison of trajectories between Participants 1 and 2 compared to the asymptomatic controls, speed could not be modelled as a covariate because all analysed gait cycles were collected at the same running speed.

For discrete, zero-dimensional scalar outcome variables (cadence (steps/minute), running speed (m·s^−1^)) and contact time (ms), descriptive statistics (means, standard deviations and mean differences) were calculated. Independent-samples *t*-tests were then used to determine whether a significant (*p* < 0.05) difference existed between the MTSS symptomatic and control group limbs. The effect size was calculated using Hedges’ g and interpreted in relation to the existing literature with conventional thresholds for standardised mean differences (0.2 small, 0.5 moderate, 0.8 large) used only as approximate guides for discrete cross-sectional comparisons [24]. Discrete outcome variables were analysed using SPSS 29.0 for Windows (IBM Inc., Armonk, NY, USA).

## 3. Results

**Figure 1:**
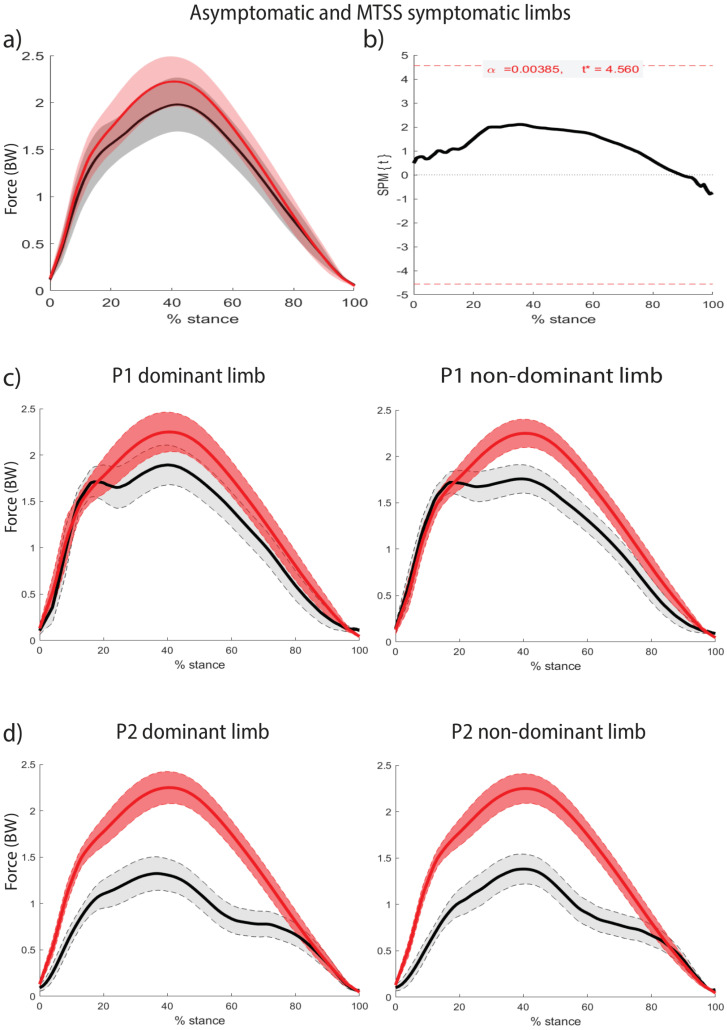
(**a**) Mean and standard deviation (SD) normal force profiles for asymptomatic limbs (mean = red line, SD = shaded red) and MTSS symptomatic limbs (mean = black line, SD = grey). (**b**) Two-sample *t*-test statistic SPM {t} for asymptomatic and MTSS symptomatic force profiles. t* = critical threshold required to surpass to meet significance. (**c**) Mean and 95% confidence interval (CI) force profile for the dominant and non-dominant limbs of Participant 1 (mean = black line, 95% CI = grey) and asymptomatic limbs (mean = red line, 95% CI = shaded red). (**d**) Mean and 95% CI force profile for the dominant and non-dominant limbs of Participant 2 (mean = black line, 95% CI = grey) and asymptomatic limbs (mean = red line, 95% CI = shaded red). Forces normalised to body weight (BW).

**Figure 2:**
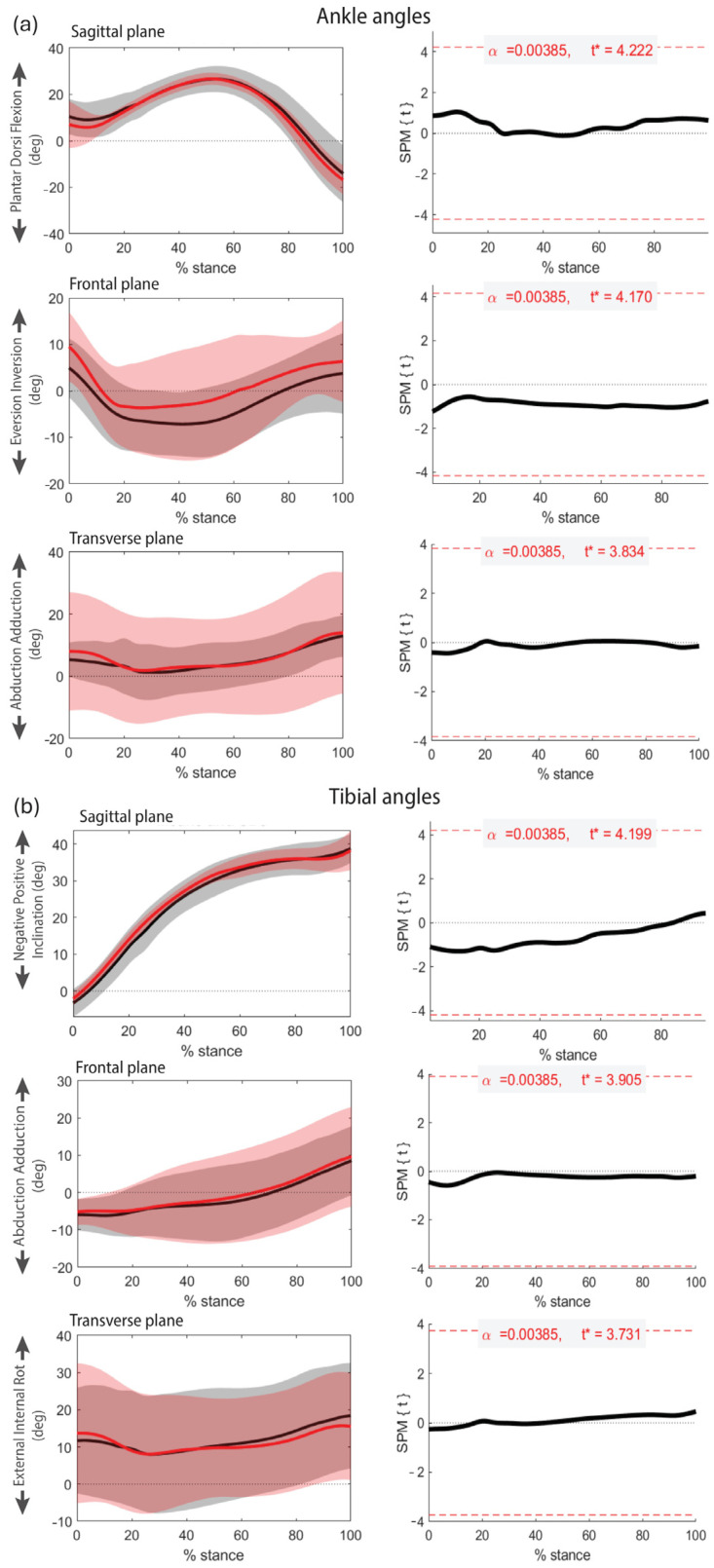

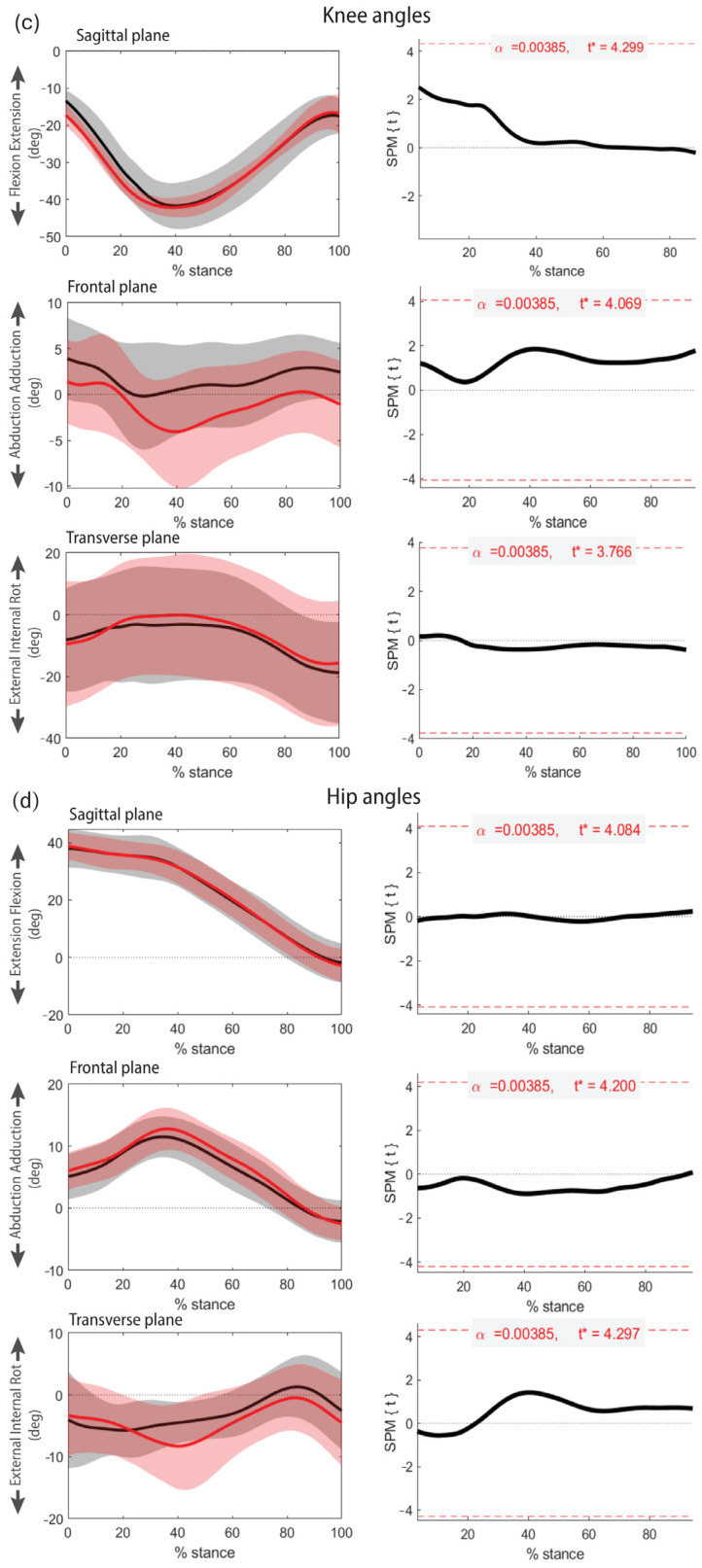
Mean and standard deviation (SD) joint angles ((**a**) = ankle, (**b**) = tibial, (**c**) = knee, (**d**) = hip) for asymptomatic limbs (mean = red line, SD = shaded red) and MTSS symptomatic limbs (mean = black line, SD = grey). Two-sample *t*-test SPM {t} statistics are also shown on the right with the critical threshold denoted by t*.

Descriptive statistics for the spatiotemporal outcome variables derived for the MTSS symptomatic limbs and the control limbs are shown in Table 2. Compared with the asymptomatic controls, the MTSS symptomatic participants had a significantly slower running speed and increased foot-ground contact time. No statistically significant differences were found in the SPM trajectories of the normal force or in the 3D kinematics of the tibia and ankle, knee or hip joints between the MTSS symptomatic limbs and their matched controls (see Figure 1 and Figure 2). For our primary analysis, across all 13 trajectories, no suprathreshold effects were detected with group-mean trajectories remaining comparable throughout the entire stance phase. However, for our secondary analysis, suprathreshold clusters were identified in the transverse plane for both the knee (approximately 5–10% of stance; cluster-level *p* = 0.0036) and the tibia, which occurred at the lower bound of the critical threshold (cluster-level *p* = 0.0032) (see Supplementary Figure S1).

Descriptive statistics for the spatiotemporal outcome variables, comparing the two participants who developed MTSS following baseline testing (Participants 1 and 2) with asymptomatic control limbs, are presented in Table 3. Compared to the controls, Participants 1 and 2 displayed substantially different, although divergent, running speeds and corresponding foot-ground contact times: Participant 1 ran substantially faster, while Participant 2 ran considerably slower, with foot-ground contact times reflecting these speed differences.

**Figure 3:**
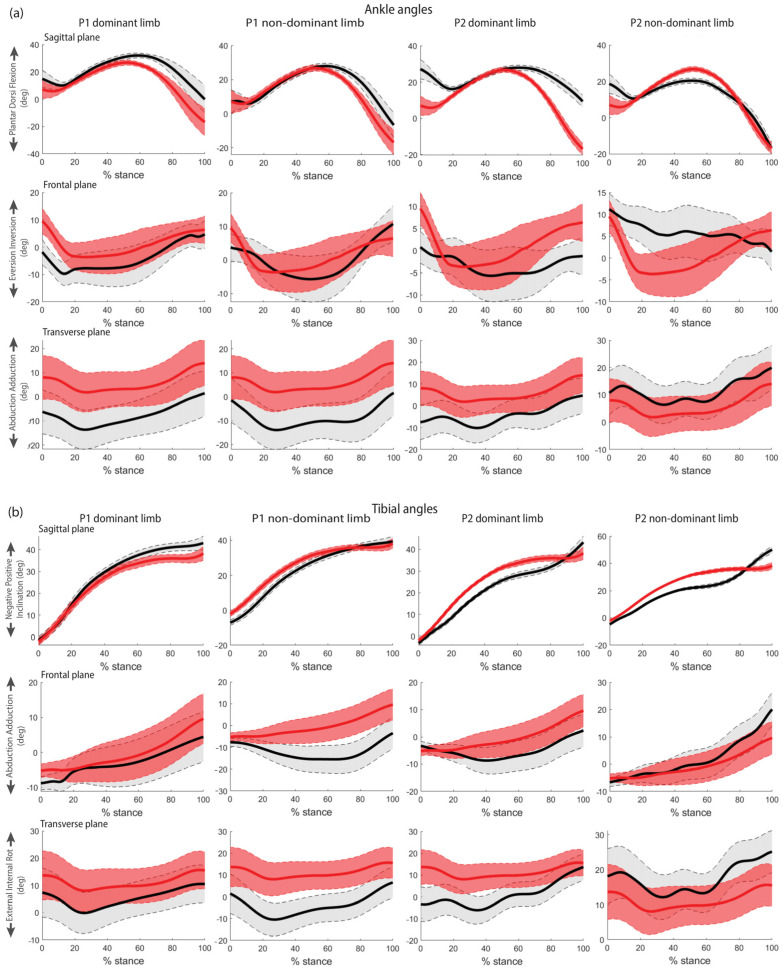

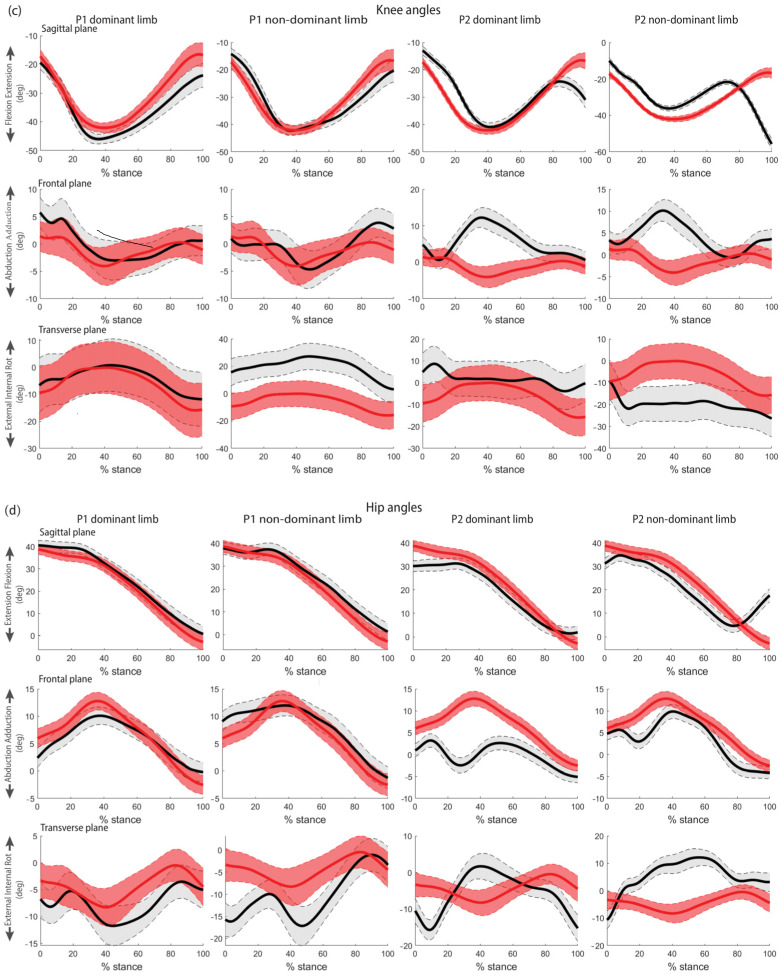
Mean and 95% confidence interval (CI) joint angles ((**a**) = ankle, (**b**) = tibial, (**c**) = knee, (**d**) = hip) for asymptomatic limbs (mean = red line, SD = shaded red) and dominant and non-dominant limbs of Participants 1 and 2 (mean = black line, SD = grey).

The mean force trajectories and 95% confidence intervals were non-overlapping for three out of the four case study limbs, in which Participants 1 and 2 generated substantially less normal force during the propulsive phase of stance than non-injured runners (see Figure 1). Descriptive analyses of the kinematic trajectories revealed non-overlapping 95% confidence intervals in the sagittal plane at the ankle joint in three of four limbs, in which Participants 1 and 2 displayed reduced ankle plantar flexion during the propulsive period of stance. Both participants also displayed a more vertical tibia during the absorption and early propulsive periods of stance. Opposing hip-rotation strategies were identified between Participants 1 and 2, which were also substantially different from those of the control group. That is, compared with the control group, Participant 1 displayed greater external rotation at the hip, whereas Participant 2 displayed greater internal rotation. Furthermore, compared to the control group, Participant 2 exhibited less knee flexion during the absorption phase of gait, greater knee flexion during the propulsive phase, and greater bilateral knee adduction and hip abduction (see Figure 3).

## 4. Discussion

Although active peak force and lower-limb kinematics have previously been reported to be associated with tibial bone stress injuries, prospective research and continuous analysis techniques are required to better identify kinetic or kinematic risk factors associated with MTSS. In this study, our primary analysis of retrospective data demonstrated that long-distance runners who continued to train despite suffering MTSS symptoms showed no significant differences in cadence, force or 3D kinematic trajectories of the tibia, ankle, knee or hip joints during stance compared with their asymptomatic matched controls. However, these null findings should be interpreted cautiously, as the relatively small sample size may have limited statistical power to detect subtle but clinically meaningful biomechanical differences in the case-control comparisons, increasing the risk of a Type II error. Notably, significant differences in running speed were identified. To explore the potential influence of running speed on the observed findings, post hoc SPM1D regression analyses were performed. These analyses revealed suprathreshold clusters in the transverse plane of the knee and tibia during the absorption phase of running.

Relative to asymptomatic controls, the two participants who were prospectively followed and developed MTSS after baseline testing displayed lower normalised active peak force bilaterally, a more vertical tibia during absorption, and early propulsive periods of gait and less plantar flexion during propulsion. The implications of these findings are discussed below.

### 4.1. Case-Control Comparison

Reduced running speed and increased foot–ground contact times in the MTSS group were accompanied by large effect sizes, suggesting clinically meaningful between-group differences. However, the observed variability in running speed suggests inconsistent individual responses. The between-group differences in running speed represent a potential confounding variable. Despite this, the significant speed × group interaction indicates that running speed influenced transverse-plane knee and tibial kinematics differently between MTSS runners and controls. In contrast, no such interaction was observed for sagittal or frontal plane kinematics or normal force profiles. These findings should be interpreted with caution due to the small sample size, which may limit the stability of variance estimates within the SPM1D general linear model regression framework. Consequently, interpretation focused on the presence and timing of the speed × group interaction rather than the magnitude of the regression slopes. The slower self-selected running speed identified in this paper aligns with previous research, in which significantly higher visual analogue pain scale scores at rest were associated with a slower self-selected running speed in 14 MTSS symptomatic individuals compared with 19 asymptomatic controls [25]. Furthermore, Boer et al. [26] found that long-distance runners with a history of MTSS ran significantly slower than their asymptomatic counterparts and that the prevalence of a history of MTSS was significantly lower for every 1 km/h increase in running speed (*p* = 0.0381). The slower running speeds and longer contact time in the current study may reflect either a strategy to reduce tibial loading by reducing instantaneous force while increasing load duration per step [2,3] or a limitation in propulsive capacity. Previous research has shown that compared to asymptomatic controls, MTSS symptomatic individuals display deficits in plantar flexor endurance capacity and maximal voluntary isometric contraction strength of the soleus, flexor hallucis longus and peroneal muscles [27,28,29]. Such impairments may limit the ability to generate and sustain propulsive force during running. Consequently, slower self-selected running speeds could reflect pain-related adaptations or a reduced capacity to generate efficient propulsive force.

### 4.2. Case-Study Comparison

Supporting the notion of a reduced propulsive capacity, during baseline testing, Participants 1 and 2 developed MTSS despite displaying substantially lower normalised force profiles during stance than the asymptomatic controls. Developing MTSS, despite exhibiting lower normal forces and, therefore, potentially lower tibial bone loads than the asymptomatic controls, suggests that the threshold for tibial bone load above which MTSS develops may be unique to each individual. Potentially, the greater propulsive force displayed by the asymptomatic runners could contribute to different loading patterns with a possible shift toward relatively more compressive loading at the posteromedial tibia, which is a loading mode that bone may better tolerate [30]. Several studies support this theory, including an in vivo study by Yang et al. [5] and an inverse dynamics study by Rice et al. [6], which showed that posterior compressive stresses increase with gait speed. Furthermore, an in vivo study by Milgrom et al. [31] found that the tension strain and strain rate increased by 29% and 11%, respectively, at the medial tibia after a fatiguing running and marching protocol, during which a significant decrease in peak gastrocnemius isokinetic torque was also observed. However, as tibial bone loading patterns cannot be inferred from the current data, further prospective studies assessing tibial bone loads in individuals who develop MTSS are required to confirm or refute this notion.

Consistent with this reduced propulsive capacity, Participant 2 self-selected a substantially slower running speed than the asymptomatic controls, which likely contributed to her lower active peak force during stance. The slower running speed may be associated with her history of MTSS three years before her baseline assessment. However, whether reduced running speed persists following the recovery from MTSS remains unclear and warrants further prospective research.

At a kinematic level, the deficits in active force production displayed by Participants 1 and 2 are reflected in reduced ankle plantar flexion during propulsion and a more vertical tibia during the absorption and early propulsive phase of stance in all but one limb that developed MTSS. However, forward tibial inclination at toe-off was not substantially different from that of the control participants. At an individual level, the greater bilateral knee flexion displayed by Participant 2 during toe-off, compared to the asymptomatic controls, is likely associated with her running speed of 1.8 m·s^−1^ and the absence of a flight phase (grounded running). In contrast, the control participants ran at 3.6 m·s^−1^, which is a speed typically characterised by an aerial phase. Therefore, a comparison of the kinematic and force profiles between Participant 2 and the controls should be interpreted cautiously. Such characteristics are reported to be a feature of economical female running gait [32], likely by allowing the leg extensor muscles to operate at a more favourable position on the force–length curve and by achieving higher gear ratios [33]. However, the magnitude of knee flexion displayed by Participant 2 substantially exceeds the values reported by Moore et al. [32].

The opposing hip-rotation strategies observed between Participants 1 and 2 could be associated with MTSS risk, sex-specific biomechanical characteristics or a combination of both. For example, Newman et al. [34] reported that an increased passive hip external range of motion in males was associated with an elevated risk of MTSS development, although the underlying mechanistic link remains unclear. In contrast, recreational female runners have been reported to exhibit greater internal hip rotation than male runners, which could be related to sex-specific differences in Q-angle or kinematic patterns [35].

### 4.3. Clinical Implications

From a clinical perspective, these findings suggest that reduced running speed and lower stance phase force do not necessarily prevent MTSS onset. As such, a holistic approach is needed to reduce the risk of MTSS development rather than simple force- or speed-based modifications. Practitioners should, therefore, exercise caution when using peak force metrics to infer MTSS risk. The observed reductions in plantar flexion kinematics and force generation in runners who developed MTSS highlight the potential importance of plantar flexor strength and endurance training in mitigating MTSS development risk. Interventions aimed at improving plantar flexion force-generating capacity should be individualised and progressed cautiously, with close monitoring of symptoms and training load, rather than assuming that slower running or reduced force production inherently lowers the risk of MTSS development.

### 4.4. Limitations

To our knowledge, this is the first paper to prospectively assess continuous multiplanar running kinematics and normal forces in long-distance runners with MTSS compared to asymptomatic controls. Nevertheless, this paper has several limitations. Firstly, to enable participants to achieve a gait representative of their 10 km race pace, running speed was not controlled, and participants were matched for running distance rather than running speed. Therefore, differences in self-selected running velocities may have influenced the measured kinematic and force variables. Although SPM1D regression analyses were used to examine the influence of self-selected running speed in the case-control comparisons, this approach could not be applied to the case study analyses because all steps for Participants 1 and 2 were collected at an identical running speed, precluding any statistical estimation of a speed-dependent effect due to the absence of variability.

Second, to capture force data for every step during the running trials, Pedar-X pressure measuring insoles (Novelgmbh, Munich, Germany) were used. Although these insoles provide valid [36] and reliable measures of normal force (N) [37], they have a limited sampling rate (100 Hz), which may have influenced the peak force data. However, this limitation is partially mitigated by averaging 10 stance phases per participant (*n* = 240 total phases).

Third, for bilateral MTSS cases, the most symptomatic limb was selected post hoc, potentially introducing selection bias. Finally, the small sample sizes in both the case-control analysis and case study reduce the generalisability of our findings, and we acknowledge that conclusions drawn from case-control comparisons cannot determine whether the between-group differences were causes or effects of MTSS development.

## 5. Conclusions

Compared with well-matched controls, runners with MTSS exhibited slower running speeds but no significant differences in normalised force profiles or leg kinematics across stance. From our exploratory analysis of prospective observations, the two runners who developed MTSS during the study displayed lower normal forces, reduced plantar flexion and a more vertical tibia compared to asymptomatic controls, suggesting that lower force and a more upright tibial angle did not preclude the development of MTSS in these cases. Instead, the bone load threshold for MTSS development may be individual, and, therefore, peak force during stance may have limited utility as a standalone screening tool for MTSS risk. Collectively, the findings from our case-control and case-study suggest that plantar flexion function may be implicated in the development of MTSS and that running speed deficits may persist during ongoing episodes. However, larger prospective studies should investigate ankle function, propulsive force capacity and MTSS development in runners matched for habitual running speed given that differences in self-selected speeds may confound the interpretation of kinematic and kinetic outcomes.

## Figures and Tables

**Table 1:** Characteristics of the MTSS symptomatic (*n* = 11) and matched control participants (*n* = 11) and of the participants who developed MTSS (*n* = 2).

Variables	MTSS Symptomatic	Control	P1	P2
Sex (female/male)	8/3 ^#^	8/3 ^#^	Male	Female
Age (years)	32.9 ± 9.2	32.5 ± 9.4	26.3	47.5
Height (cm)	172 ± 5.1	170 ± 8.5	192	159
Mass (kg)	68.3 ± 6.3	66.1 ± 6.2	78.8	71.8
Body mass index (kg/m^2^)	23.2 ± 2.3	22.8 ± 2.0	21.5	28.4
Foot strike pattern, RF, MF or FF	10/0/1 ^#^	8/3/0 ^#^	RF	RF
Weekly training distance (km)	32.3 ± 12.9	34.1 ± 12.2	25	23

MTSS symptomatic and control values are mean ± standard deviation, except for ^#^, which is a count. FF = forefoot, MF = midfoot, P1 = participant 1, P2 = participant 2, RF = rearfoot, weekly training distance (km) = weekly average over the previous six months.

**Table 2:** Spatiotemporal outcome variables for 11 MTSS symptomatic and 11 asymptomatic control limbs.

Variables	MTSS Symptomatic Limbs	Asymptomatic Control Limbs	Mean Difference	Hedges’ g	*p*-Value
Cadence (steps/minute)	164.7 (14.3)	169.1 (8.4)	−4.4	0.36	0.393
Running speed (m·s^−1^)	3.0 (0.6)	3.6 (0.5)	−0.6	1.09	0.034 *
Contact time (ms)	0.27 (0.02)	0.24 (0.02)	0.04	−1.44	<0.001 *

Values are mean (±SD), * *p* < 0.05, Mean difference = MTSS symptomatic limbs − Control.

**Table 3:** Spatiotemporal outcome variables for the two participants who developed MTSS after baseline testing (Participants 1 and 2) compared to 11 asymptomatic control limbs.

Variables	P1	Mean Difference	P2	Mean Difference
Cadence (bilateral steps/minute)	168	−1.1	168	−1.1
Running speed (m·s^−1^)	4.0	0.4	1.8	−1.8
Contact time (bilateral, ms)	0.23	−0.01	0.40	0.16

Mean difference = Participant − asymptomatic control limbs (Table 2), P1 = Participant 1, P2 = Participant 2.

**Figure A1:**
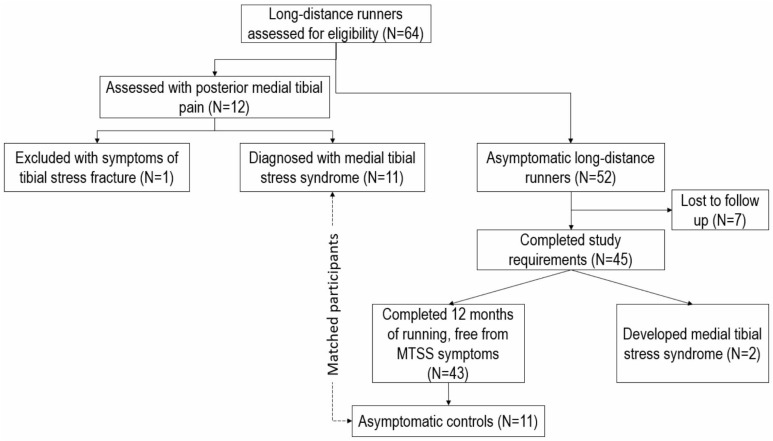
Flow chart of participants included in the study.

## Data Availability

The data presented in this study are available on request from the corresponding author due to participants privacy.

## Supplementary Materials

The following supporting information can be downloaded at https://www.mdpi.com/article/10.3390/jfmk11020214/s1. Supplementary Figure S1. Statistical parametric mapping regression results for the speed × group interaction across the stance phase. Panel a = ankle and tibial kinematics, panel b = knee and hip kinematics, and panel c = normal force. t* = critical threshold required to surpass to meet significance with supra-threshold clusters denoting statistically significant interaction effects.
