# Supplementary figures and images for: Are the Forces and Lower Limb Kinematics Displayed During Running Associated with Medial Tibial Stress Syndrome? A Case-Control and Case Study
Source: J Funct Morphol Kinesiol. 2026 May 28;11(2):214. doi: 10.3390/jfmk11020214 (PMC13302312; doi:10.3390/jfmk11020214)

# Ankle angles

# Tibial angles

(a)

Sagittal plane

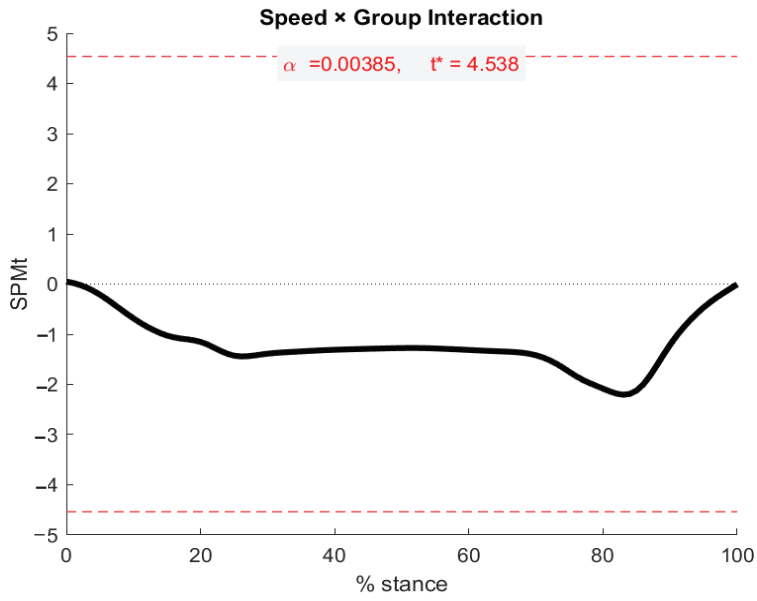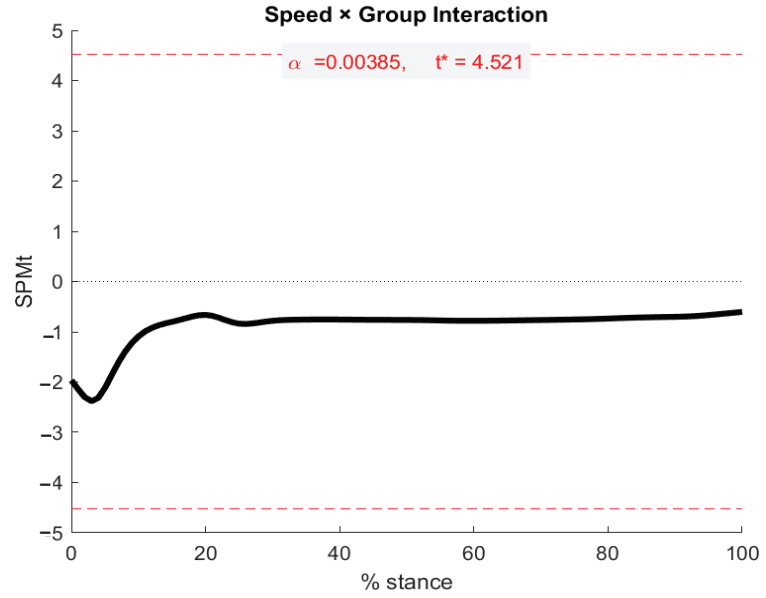

Frontal plane

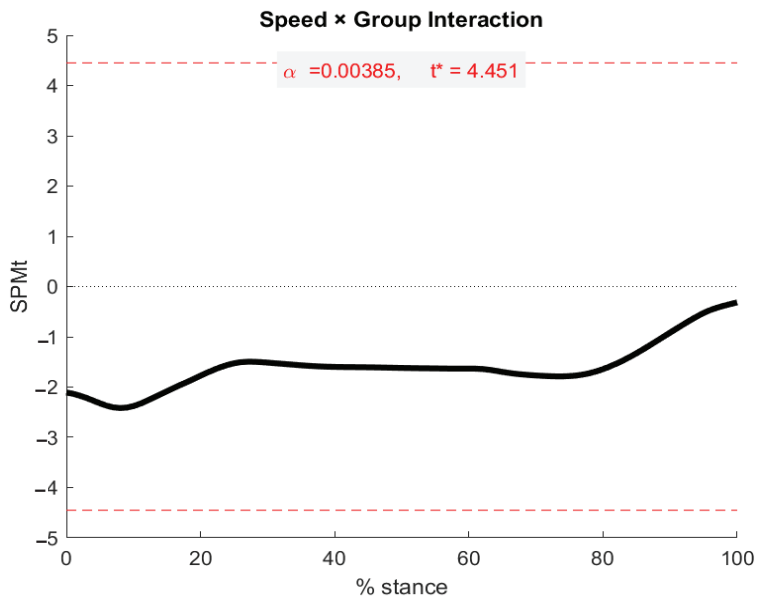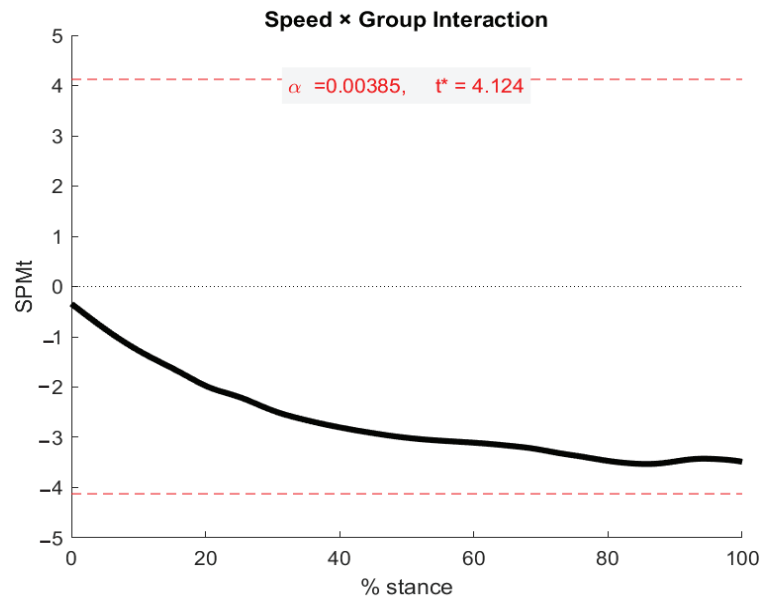

Transverse plane

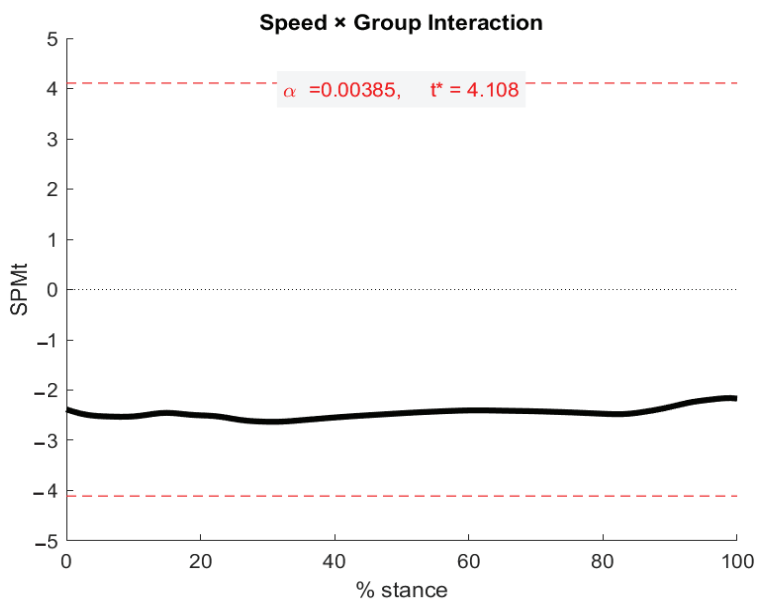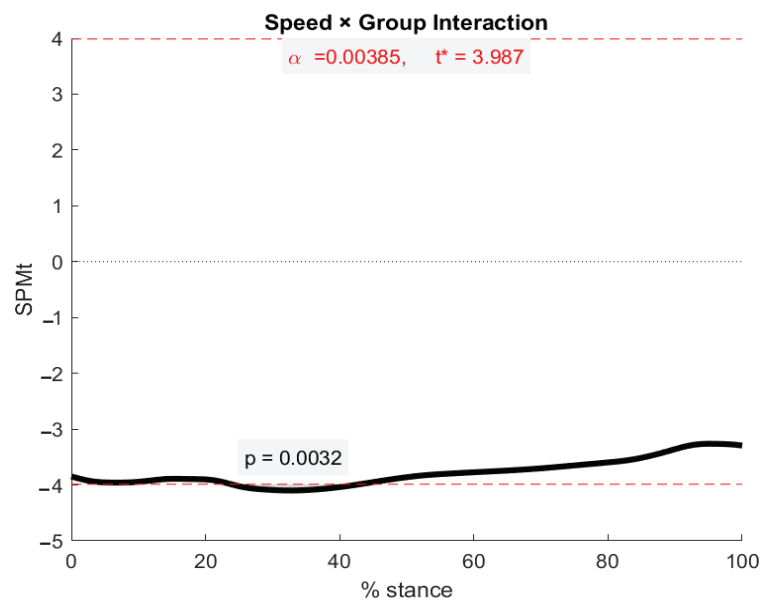

Supplement: Supplementary file 1 [file jfmk-11-00214-s001.zip › Supplementary Figure S1 a SPM regression 26-05-26 ankle tibia.pdf]

(b) Knee angles

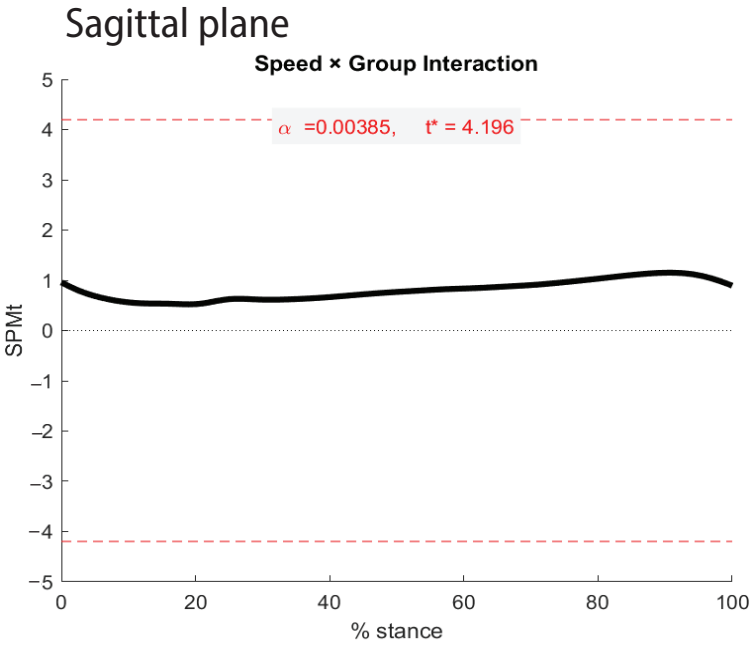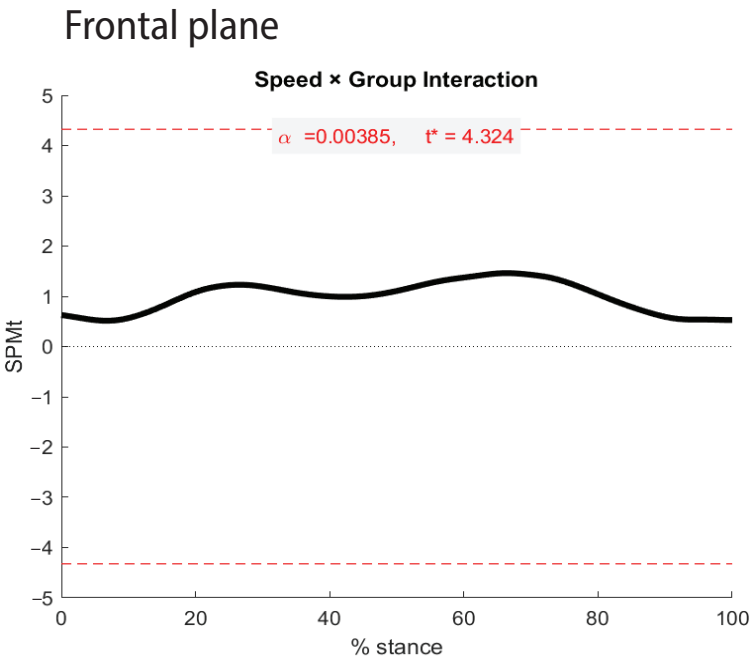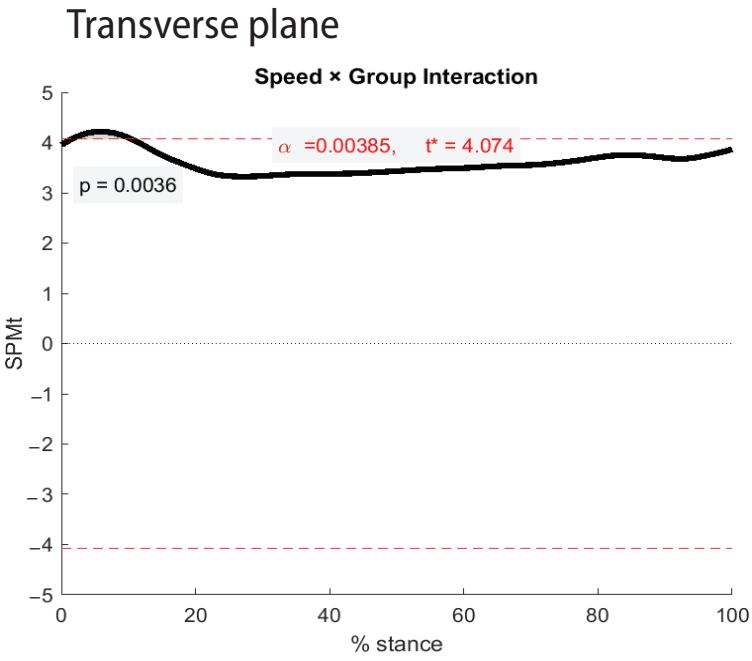

Hip angles

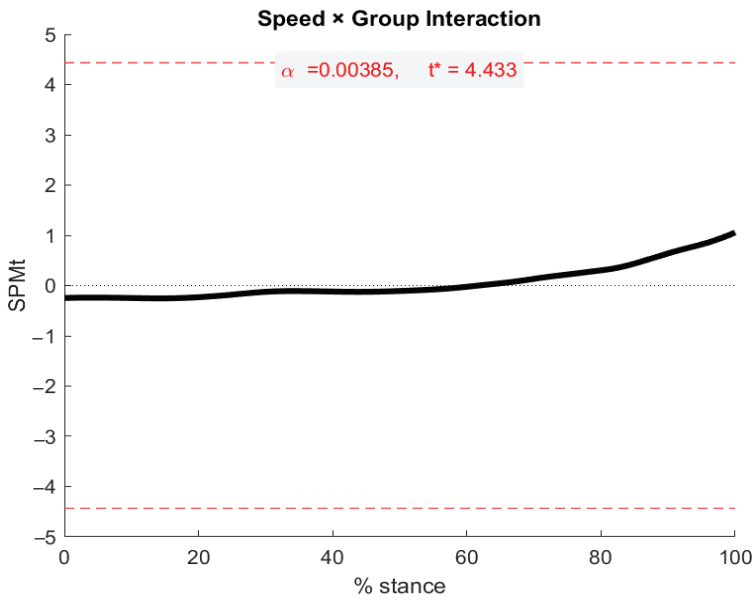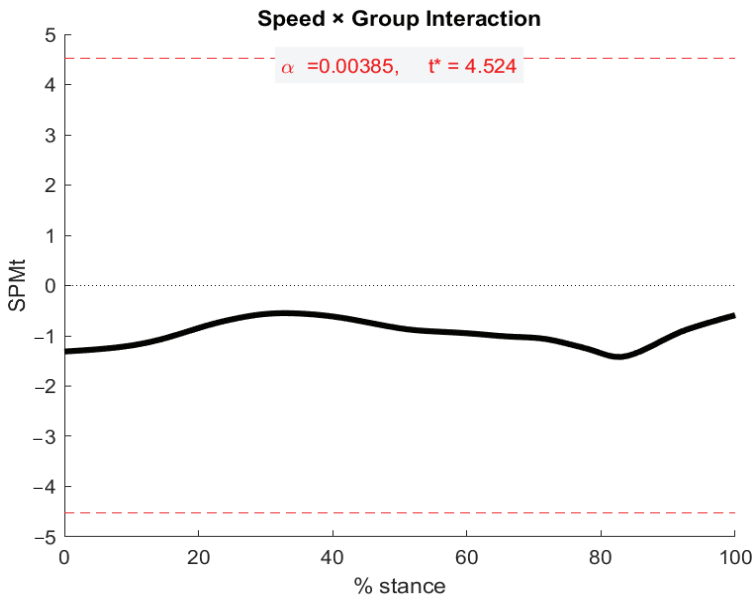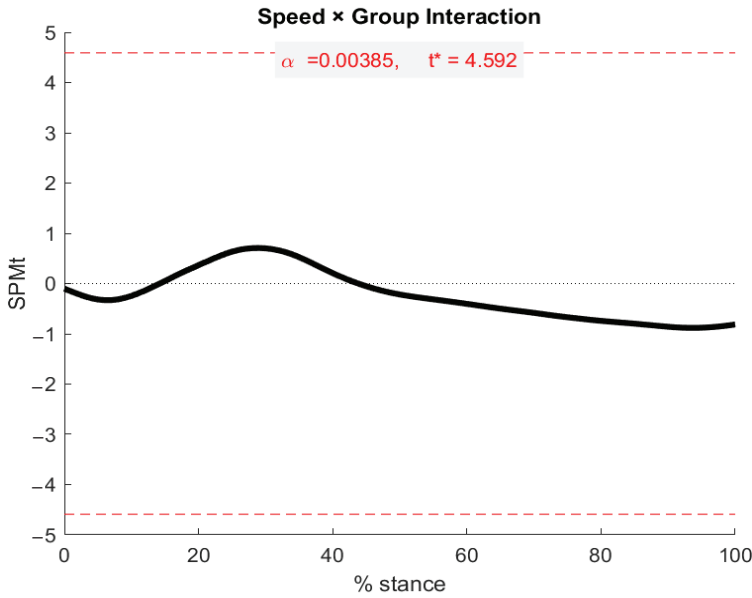

Supplement: Supplementary file 1 [file jfmk-11-00214-s001.zip › Supplementary Figure S1 b SPM Regression 26-05-26 knee hip.pdf]

(c)

# Normal force

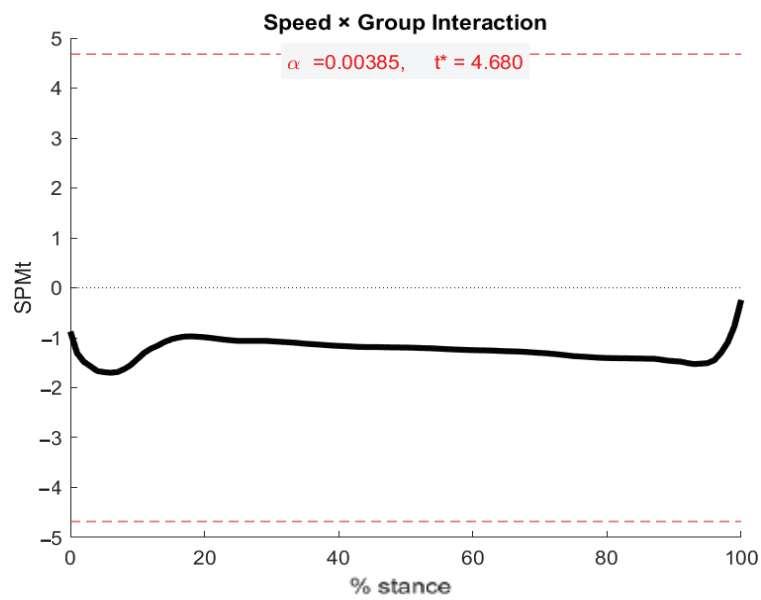

Supplement: Supplementary file 1 [file jfmk-11-00214-s001.zip › Supplementary Figure S1 c SPM regression 26-05-26 force.pdf]
